# The role of galectin-4 in physiology and diseases

**DOI:** 10.1007/s13238-016-0262-9

**Published:** 2016-03-26

**Authors:** Zhan-Qi Cao, Xiu-Li Guo

**Affiliations:** Department of Pharmacology, School of Pharmaceutical Sciences, Shandong University, Jinan, 50012 China

**Keywords:** galectin-4, lipid raft, crosslinker, intestinal inflammation, tumor progression

## Abstract

Galectin-4, a tandem repeat member of the** β**-galactoside-binding proteins, possesses two carbohydrate-recognition domains (CRD) in a single peptide chain. This lectin is mostly expressed in epithelial cells of the intestinal tract and secreted to the extracellular. The two domains have 40% similarity in amino acid sequence, but distinctly binding to various ligands. Just because the two domains bind to different ligands simultaneously, galectin-4 can be a crosslinker and crucial regulator in a large number of biological processes. Recent evidence shows that galectin-4 plays an important role in lipid raft stabilization, protein apical trafficking, cell adhesion, wound healing, intestinal inflammation, tumor progression, etc. This article reviews the physiological and pathological features of galectin-4 and its important role in such processes.

## INTRODUCTION

Galectins, composed of 15 members, have been identified as galactoside-binding proteins localized both intra- and extra-cellularly. The galectin family members were widely found in various bionts including vertebrates, invertebrates and even protistans (Cooper and Barondes, 1999). Based on the composition and recognition of the conserved carbohydrate-recognition domain (CRD), galectins are grouped into three subfamilies. The prototype galectins, also called monomer, contain one CRD and include galectins-1, 2, 5, 7, 10, 11, 13, 14, and 15. The tandem repeat types contain two distinct but homologous CRDs and include galectins-4, 6, 8, 9, and 12. The only chimera type (galectin-3) is defined by a C-terminal CRD and an elongated N-terminal region that induces the formation of multimers. These galectins are widely present in various types of human cells and participate in various cellular functions, such as cell proliferation, apoptosis, adhesion, signal transduction, immune response, activation of inflammatory response, and regulation of pre-mRNA splicing (Rabinovich, 1999; Cooper, 2002; Danguy et al., 2002; Barrow et al., 2013).

Galectin-4 was first isolated as a 17 kDa protein in the extract of rat small intestine (Leffler et al., 1989). Subsequently, it was identified as a proteolytic fragment of a larger 36 kDa protein by gene cloning (Oda et al., 1993), and found intracellularly, on the cell surface, and in circulation. *In vitro*, intracellular galectin-4 regulates cell proliferation, apoptosis and differentiation, whereas extracellular galectin-4 mediates intercellular adhesion (Huflejt et al., 1997; Huflejt and Leffler, 2003). Because of no signal sequence for endoplasmatic reticulum transport, the presence of galectin-4 on the cell surface is a consequence of secretion via non-classical pathway. This protein specifically binds to β-galactosides through the two structurally conserved CRDs. As is shown, widely known natural ligands of galectin-4 are human blood group antigens, glycoproteins, mucin like membrane MUC1, glycosphingolipids, and sulfated cholesterol. In addition to β-galactosides, galectin-4 could also bind to sulfate. The presence of 3′-sulfation in galactose and lactose was proved to increase the binding affinity to galectin-4 (Ideo et al., 2002; Vokhmyanina et al., 2012). Because of the bivalent or multivalent structure and affinity to above mentioned ligands, galectin-4 plays a crucial role in biochemical regulation and tumor development and progression.

Recently, many studies have been performed with the aim to clarify the interaction between galectin-4 and physiological regulation, intestinal inflammation and cancer. For example, galectin-4 was identified as a marker in detergent-resistant membranes (DRMs) and a crucial component to stabilize lipid rafts (Delacour et al., 2005). And a contradictory role of galectin-4 on intestinal inflammation was stated to exacerbate (Hokama et al., 2004; Hokama et al., 2008) or ameliorate inflammation (Paclik et al., 2008a). Further studies are required to appraise the influence of galectin-4 in intestinal inflammation. Moreover, galectin-4 has also showed contradictory roles in cancer which may depend on the types of cancer (Rechreche et al., 1997; Belo et al., 2013; Maftouh et al., 2014; Cai et al., 2014; Hayashi et al., 2013; Hippo et al., 2001). This review summarizes the recent progress in understanding the relationship between the structure and the function of galectin-4 in physiological and pathological processes.

## THE MOLECULAR STRUCTURE OF GALECTIN-4 AND BINDING AFFINITY TO LIGANDS

As a tandem-repeat, human galectin-4 contains two distinct but homologous domains: CRD1 (N-terminal) and CRD2 (C-terminal). These two CRDs have a calculated molecular weight of 16–17 kDa. Each of the CRDs consists of about 130 residues which share 40% sequence identity, and they are connected by a linker region (Oda et al., 1993; Jiang et al., 1999). This linker region, which is composed of about 30 residues, is rich in proline and glycine, and is sensitive to tissue proteases (Rustiguel et al., 2015). It is believed that galectin-4 can be a natural crosslinker because of its ability to crosslink two distinct types of ligands (Brewer, 2002). The carbohydrate binding specificities of the two CRDs are quite different and would be expected to show preference for different sets of ligands.

Two CRDs in galectin-4 bind lactose with similar affinity, but their preferences for other glycosphingolipids, oligosaccharides, and glycoprotein are distinctly different. For example, the affinity of CRD2 (*K*_*D*_*=* 2.0 × 10^−6^ mol/L) toward SO_3_^−^→3core 1-O-Bn oligosaccharide was higher than that of CRD1 (*K*_*D*_ =2.3 × 10^−5^ mol/L), whereas CRD1 (*K*_*D*_*=* 8.9 × 10^−5^ mol/L) showed higher affinity toward Fucα1→2-Galβ1→3GlcNAcβ1→3Galβ1→4Glc than that of CRD2 (*K*_*D*_ = 1.2 × 10^−4^ mol/L) (Ideo et al., 2005). CRD2 also showed higher affinity toward 3-O-sulfated glycosphingolipids than that of CRD1 (Ideo et al., 2005). Human CRD2 showed higher binding affinity toward saccharides expressed on ABO(H) blood group antigens than that exhibited by CRD1 (Vokhmyanina et al., 2012). Oligosaccharide binding profiles showed that the CRD2 in mouse galectin-4 had a high affinity and specificity for A type-2 α-linked N-acetylgalactosamine (α-GalNAc) structures, while the CRD1 domain showed a broader affinity compared to CRD2 (Marková et al., 2006). In addition to saccharides, the drastic difference is that CRD1 is able to bind to cholesterol-3-sulfate while CRD2 is incapable (Ideo et al., 2007). An Arg45 in CRD1 was identified as a core for this sulfate recognition, while none of the amino acids in CRD2 domain was identified to directly interact with sulfate groups (Ideo et al., 2007; Bum-Erdene et al. 2015). Moreover, a peptide YVQI in CRD2 was confirmed to bind to Src kinases, therefore regulating the phosphorylation and externalization of galectin-4 (Ideo et al., 2013). However, there is very limited information about the key binding partners underlying binding specificities and key differences for the two domains.

## THE PHYSIOLOGICAL FUNCTIONS OF GALECTIN-4

### Galectin-4 enhances the stabilization of lipid raft

Lipid rafts are characterized as heterogeneous and liquid ordered microdomains in the brush border membrane of small intestinal enterocytes (Pike, 2005). Rafts are small sphingolipids-rich and cholesterol-rich platforms in the outer exoplasmic leaflet of the lipid bilayer (Simons and Ehehalt, 2002). Lipid rafts are also known as DRMs because of their common ability to resist solubilization with detergents in various cell membranes (London and Brown, 2000). As a divalent galectin, galectin-4 was identified as a major component of detergent-insoluble complexes prepared from the small intestine (Danielsen and van Deurs, 1997). In addition, galectin-4 shows the ability to act as a cross-linker, thus indicating that galectin-4 might play a role in lipid rafts stabilization.

**Figure 1:**
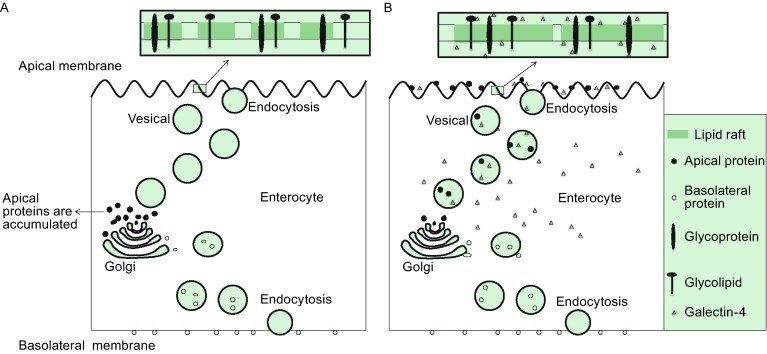
**Galectin-4 participates in apical proteins trafficking and lipid raft stabilization**. (A) In the absence of galectin-4, the apical proteins are accumulated intracellularly. Lipid rafts are small in the plasma membrane, containing only a subset of glycoproteins and glycolipids. (B) In the presence of galectin-4, apical proteins are trafficked to the apical plasma membrane. Lipid rafts are capable of clustering based on galectin-4 crosslinking to glycoproteins and glycolipids in DRMs

Galectin-4 facilitates stabilization of lipid rafts through formation of homogenous lattices with some glycoproteins and glycolipids (Brewer et al., 2002). Galectin-4 could cross-link a broad range of glycolipids and various brush border proteins on the surface of enterocytes, and form cluster and lattices with them. The externalized galectin-4 will stay at the cell surface and almost specially localized to the brush border (Danielsen and Hansen, 2008), and target to the outer exoplasmic leaflet of the brush border where it specially associates with lipid rafts and other enzymes, mainly aminopeptidase N and sucrase-isomaltase (Danielsen and van Deurs, 1997). These enzymes are frequently cleaved and released into the gut lumen by exposing to pancreatic proteinases and lipases. Galectin-4 was reported to protect the brush border enzymes from solubilization by binding simultaneously to membrane glycolipids and enzymes, thus eventually protecting cleaved enzymes releasing into the gut lumen (Danielsen and Hansen, 2008). Generally speaking, at the presence of galectin-4, the lipid rafts are capable of clustering, which means that these proteins and glycolipids are stabilized in stationary microdomains (Fig. 1B). Thus, galectin-4 can be characterized as an organizer/stabilizer within microvillar lipid rafts.

### Galectin-4 participates in apical trafficking

The surface of enterocyte is divided into basolateral domains and apical with distinct compositions and functions. The apical surface contains the proteins required for organ-specific functions while the basolateral surface expresses adhesion molecules and receptors, which creates an asymmetric structure. This asymmetric structure implies a polarized protein targeting which is determined by apical and basolateral sorting signals (Mostov et al., 2000). The sorting signals are capable of deciding which vesicle the proteins could move into, then guiding these proteins to the apical or basolateral membrane. The basolateral targeting signals are regulated by tyrosine residues existed in the cytoplasmic domain of proteins (Matter and Mellman, 1994). However, signals for apical trafficking seem more diversified because various signals have been found in cytoplasmic, transmembrane or extracellular domains (Schuck and Simons 2004).

Surprisingly, in galectin-4-knockdown HT-29 5M12 cells, the apical proteins, including mucin-1 (MUC1), sialyltransferase, dipeptidylpeptidase-IV (DPP-IV), nonspecific cross-reacting antigen (NCA), carcino-embryonic antigen (CEA), and the glycosyl-phosphatidylinositol (GPI)-anchored complement regulatory protein (CD59), are found depleted in DRMs (Delacour et al., 2005, Stechly et al., 2009), which implies that galectin-4 may function as the carrier of glycoproteins trafficking. Complex-type N-glycans have been shown to function as an apical sorting signal and it’s recognition by galectin-4 has emerged as a novel apical sorting mechanism (Morelle et al., 2009). Complex-type N-glycans, which are rich in DRMs, are composed of branched N-acetyllactosamine (ligand of galectin-4) and hybrid-type structures. Moreover, it has been proved that complex-type N-glycans could enhance the binding affinity of galectins to glycoproteins (Hirabayashi et al., 2002). Interestingly, if N-glycans do not form complex-type, the apical glycoproteins would be delivered to the basolateral membrane in HT-29 5M12 cells (Stechly et al., 2009).

Glycoproteins are sorted in the trans-Golgi network (TGN) into carriers that take them directly to the apical side in the presence of galectin-4 (Fig. 1B), while apical proteins accumulate intracellularly in the absence of galectin-4 (Fig. 1A). The delivery of galectin-4 at a post-Golgi level is required for the recruitment of glycoproteins within lipid rafts and their apical trafficking (Stechly et al., 2009). In addition, galectin-4 was also found in post-Golgi carrier vesicles to meet the newly synthesized apical glycoproteins (Delacour et al., 2005). Because of the special affinity to glycoproteins, galectin-4 may act as a tractor to pull them into the vesicles. In the process of apical trafficking, an apical endocytic-recycling pathway of galectin-4 is required, that is to say, galectin-4 endocytosed into cells to transport glycoproteins back to apical surface (Stechly et al., 2009).

### Galectin-4 has bactericidal activity against bacteria expressing blood group antigens

The bacteria that express blood group antigen may lead to generating self-specific antibodies to these antigens, which eventually causes immunological disorder. The carbohydrate structures of ABO (H) antigens are composed of distinct monosaccharides on the terminal structures of glycans (Yamamoto et al., 1990). This suggests that the immunity toward pathogens expressing blood group antigens must has the ability to recognize carbohydrates. Galectins are multifunctional proteins that act as regulators of various biological processes via protein-glycan interactions. Notably, previous study suggests that galectin-4 and galectin-8 could recognize and bind to blood group antigens, as along with various other saccharides ligands (Stowell et al., 2010; Liu and Bevins, 2010).

CRD2 in galectin-4 can tightly bind to blood group B carbohydrates which are expressed by some bacteria on the side chains of surface lipopolysaccharides. An antigen on the surface of *Escherichiacoli* (*E. coli*) O86 is identical to human blood group B antigen, thus indicating that galectin-4 could also bind to *E. coli* O86 (Andersson et al., 1989). Once binding to bacterial surface carbohydrates, galectin-4 directly kills *E. coli* O86 via destroying membrane integrity and bacterial motility (Stowell et al., 2010). Electron microscopy data showed that the formation of surface blebs along with disruption of *E. coli* membrane, suggesting that galectin-4 might kill the bacteria through increasing the expression of defensins (Lehrer et al., 1989). Notably, although galectin-4 could recognize human blood erythrocytes, it did not destroy the membrane integrity of these cells (Stowell et al., 2010). However, similar to other innate immune effectors, the bactericidal activity of galectin-4 is not highly specific for the blood group B antigen, but also for α-1, 3-galactose, which is another surface carbohydrate that expressed by *E.coli* (Liu and Bevins, 2010). Taken together, the binding of galectin-4 with the blood group B-related antigen expressed on bacteria causes the formation of blebs, thus leading to the death of *E.coli* O86. Up to now, the mechanism of the formation of blebs is not investigated yet.

### Galectin-4 promotes intestinal wound healing

The impaired integrity of the mucosal epithelial barrier is found not only in inflammatory bowel disease (IBD) but also in other intestinal disorders, such as peptic ulcer, intestinal infections, bowel perforation, and some other diseases. Initially, cell migration is required for restitution of the impaired mucosal epithelial barrier in the intestinal lumen. The process that the epithelial cells adjacent to the injured surface move and cover the impaired area is an important initial step. Galectin-4 was shown to have the ability to enhance epithelial cell moving to this area through binding to cadherin/catenin complex at the surface of epithelial cells (Paclik et al., 2008b). However, galectin-4 mediates the intestinal epithelial migration through an unknown mechanism. Besides migration, other processes such as the proliferation, subsequent maturation and differentiation of these cells are depicted as other important step to replenish the decreased cell pool (Dignass, 2001; Wilson and Gibson, 1997). Galectin-4 was shown to enhance the expression of cyclin B1, which could consequently improve cell cycle progression (Paclik et al., 2008b). In all, galectin-4 is capable of enhancing migration and proliferation, which suggest a significant role within the intestinal tract and a possible beneficial effect toward impaired epithelial barrier in intestinal. However, the precise mechanism of galectin-4 on promoting intestinal wound healing has not been elucidated.

### Galectin-4 promotes growth of axon and myelination in neuron

As an output channel of neuron, axon is essential for nerve conduction and rapid transmission of nerve impulses. Galectin-4 in neuron is required for axon growth (Storan et al., 2004; Velasco et al., 2013). It was proved to promote the growth of axon through increasing the cluster number and size presence of neural cell adhesion molecule (NCAM) L1 in axon membrane (Velasco et al., 2013). NCAM L1, an axonal glycoprotein expressed by many postmitotic neurons, regulates neurite outgrowth, nerve conduction and branching through L1-L1 homophilic interactions (Cheng and Lemmon, 2004; Cheng et al., 2005). Galectin-4 promotes L1 membrane cluster organization through specially binding to N-acetyllactosamine (LacNAc) at branch ends of L1 N-glycans, which act as the regulator of the process of axonal transport of synaptic glycoproteins (Velasco et al., 2013). Therefore, galectin-4 is crucial for proper organization and function of L1 in central nervous system (CNS).

**Figure 2:**
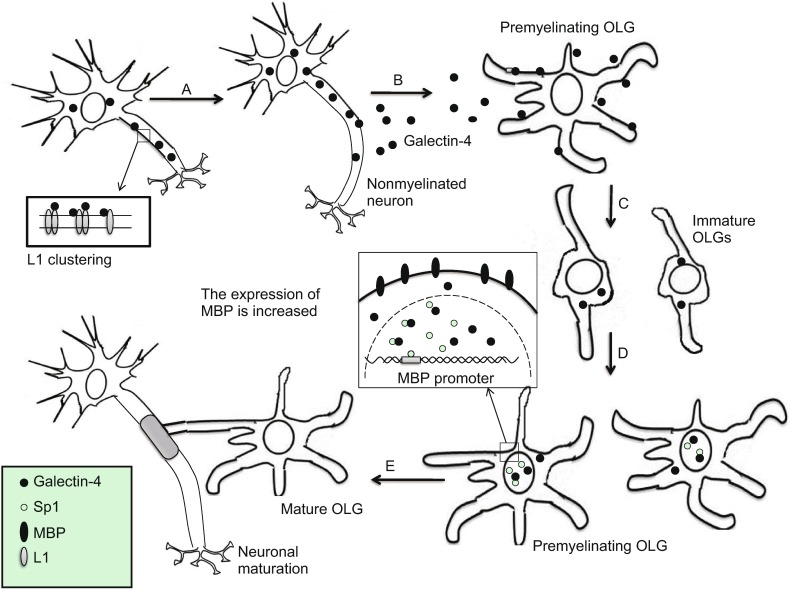
**Galectin-4 promotes growth of axon and myelination in neuron**. (A) Galectin-4 promotes outgrowth of axon through promoting L1 clustering. (B) Galectin-4 is released by nonmyelinated neurons and binds to the cell surface receptors that expressed by premyelinating OLGs. (C) Galectin-4 inhibits OLGs maturation and promotes OLGs dedifferentiation along with proliferation. (D) Immature OLGs start to differentiate. Once galectin-4 binding to the glycosylated Sp1, the stability and possibility nuclear localization of Sp1 would be increased, then leading to the upregulation of MBP gene expression. (E) Mature OLGs make the formation of myelin sheath by wrapping axons with their own cell membrane in a spiral shape

Moreover, galectin-4 also plays an important role in the regulation of myelination of axons, and its expression is downregulated at the onset of myelination (Stancic et al., 2012). Myelin, synthesized by oligodendrocytes (OLGs), is the lipid-rich membrane that specially wraps nerve axons, thereby forming a multilamellar insulating sheath, which shows plasticity and high cognitive functions in CNS (Nave, 2010). According to the role of galectin-4 in CNS, we summarized the process of myelination into three sections: 1, galectin-4 could be expressed and released by nonmyelinated neurons, and then bound to cell surface receptors expressed by premyelinating OLGs (Stancic et al., 2012). Upon binding to the receptors, galectin-4 could promote partly the dedifferentiation and proliferation of OLGs (Stancic et al., 2012). 2, galectin-4 expressed in OLGs promotes myelin basic protein (MBP) gene expression possibly through shifting from a cytoplasmic to a nuclear localization during the maturation of OLGs. MBP, located in the myelin serosal surface, is the major protein of the myelin sheath and maintains the stability of the structure and function of CNS myelin. Galectin-4 was thought to regulate the expression of MBP through alternate binding to the glycosylated moiety of transcription factor Sp1 and, then promoting the stability and possibly nuclear localization of Sp1 (Wei et al., 2007). Sp1 could activate the MBP promoter in nucleus of OLGs (Tretiakova et al., 1999; Wei et al., 2003). 3, the expression of galectin-4 was downregulated at the onset of myelination, therefore offering a condition for the differentiation and maturation of OLGs. The mature OLGs could wrap axons with their own cell membrane in a spiral shape, eventually contributing to the formation of myelin sheath. Recently, the sulfatide, which is a high-affinity ligand for galectin-4, has been considered as an inhibitor of sulfatide axon outgrowth, leading to a hypothesis that galectin-4 may regulate myelination through interaction with sulfatide (Winzeler et al., 2011). Taken together, galectin-4 in neuron acts as a novel negative regulator of OLG differentiation, and from another point of view, galectin-4 promotes the axonal myelination in CNS (Fig. 2).

## LGALS4 GENE AND REGULATION OF GALECTIN-4 EXPRESSION

Galectin-4 is coded by the single gene LGALS4 that is numbered consistently with the proteins (Mehrabian et al., 1993; Barondes et al., 1994). Human LGALS4 region locates in q13.1-13.3 on chromosome 19, while mouse LGALS4 is about 3.2 centimorgans proximal to the *apoE* gene on chromosome 7 (Gitt et al., 1998). The coding sequence of galectin-4 is specified by nine exons and the transcript length is 1 kb. The main transcriptional start site of human LGALS4 was found at position-55 nt, which is 33 bases downstream from a near consensus TATA box and its upstream promoter elements contain HNF-4, MyoD, c-Rel, HNF-3β, CAAT enhancer binding protein (C/EBP), and HFH-2 (Huflejt and Leffler, 2003).

HFH-2, HNF-4, and HNF-3β are members of the Hepatocyte Nuclear Factor 3 (HNF-3)/fork family of transcription factors (Ye et al., 1997), which contribute to the neoplastic transformation-related increases in galectin-4 mRNA expression in liver (Kondoh et al., 1999). C-Rel, a member of the NF-κB family, was aberrantly activated or expressed in human breast cancers, as well as in other solid and hematopoietic malignancies (Cogswell et al., 2000; Sovak et al., 1997). It exists either as a heterodimer or a homodimer with some NF-kB subunits and this dimmers can potently transactivate NF-kB-dependent promoters. Furthermore, c-Rel was reported to promote the formation of C/EBP-complex and then bind to the complex (Agrawal et al., 2003). Because galectin-4 is abnormally overexpressed in human breast cancers, as well as in other solid and hematopoietic malignancies, it may be a downstream product of the NF-κB. In addition, the twin single nucleotide polymorphisms (SNPs) could potentially be related to galectin-4 upregulation via deletion and insertion of new transcription factor binding sites in colorectal cancer (CRC) (Helwa, 2010).

## THE ROLE OF GALECTIN-4 IN INFLAMMATORY AND CANCER DISEASES

### Galectin-4 and intestinal inflammation

IBD is known as a chronic intestinal inflammatory condition that is characterized by two forms of intestinal inflammation: Crohn’s disease (CD) and ulcerative colitis (UC) (Podolsky, 1991). Both of the two diseases are correlated to the activation of inflammatory memory CD4^+^ T cells in the inflamed gastrointestinal tract (Xavier and Podolsky, 2007). Currently, IBD is presumed to be a result of the complex effect of genetic factors, microbial agents, humoral immunity and environmental factors. Thus, healing of the intestinal surface epithelium is regarded as a complex network of various factors. Galectin-4 is an important factor that is related to the mucosal immunity.

Galectin-4 was demonstrated to exacerbate intestinal inflammation by directly stimulating the CD4^+^ T cells to produce IL-6 on TCR mutational colitis model (Hokama et al., 2004). IL-6, a well-known inflammatory cytokine, could exacerbate intestinal inflammation in the presence of impaired mucosal barrier or injury to the mucosa. In addition, IL-6 was also confirmed to increase the expression of B-cell lymphoma-2 (Bcl-2) and B-cell lymphoma-extra large (Bcl-xl) through activating the STAT3 signal pathway, thus inhibiting the apoptosis of CD4^+^ T cells, and then leading to sustainable development of IBD (Atreya et al., 2000; Allocca et al., 2013; Waldner and Neurath, 2014). Galectin-4 may directly interact with the CD4^+^ T cells through binding to the immunological synapse, which is a specific activator of the protein kinase C (PKC) θ-associated signaling cascade in lipid raft (Hokama et al., 2004; Nagahama et al., 2008). Through activating the PKC-associated pathway, galectin-4 stimulates the production of IL-6, therefore exacerbates intestinal inflammation. However, it is not confirmed which receptor on intestinal CD4^+^ T cells specifically crosslinks with galectin-4. Lately, an inducible colitis-associated glycome (CAG), which contains an immature (nonsialylated) core-1 O-glycan expressed by CD4^+^ T cells, was identified as a ligand of galectin-4 under intestinal inflammatory conditions (Nishida et al., 2012). Thus, galectin-4 may activate the PKCθ by binding to CAG and, then contributing to exacerbation of colitis. In consistent with this, galectin-4, which shows a high affinity to immature O-glycan (Ideo et al., 2002; Blixt et al., 2004), has been shown to exacerbate an experimental chronic colitis (Hokama et al., 2004).

**Figure 3:**
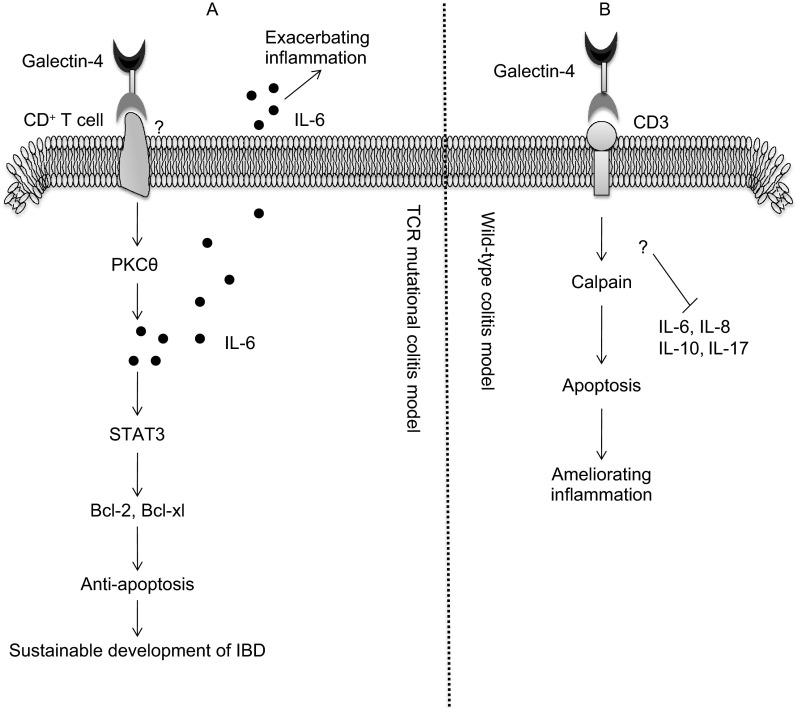
**The different molecular mechanism of galectin-4 in exacerbating or ameliorating inflammation**. (A) (Exacerbating inflammation): Through binding to the receptor (may be CAG) expressed by CD4^+^ T cells, galectin-4 stimulates the production of IL-6 on TCR mutational colitis model. IL-6 could directly exacerbate IBD and inhibiting the apoptosis of CD4^+^ T cells by activating STAT3 pathway, which eventually leading to sustainable development of IBD. (B) (Ameliorating inflammation): Galectin-4 ameliorates IBD through inducing apoptosis of T cell and reducing the secretion of inflammatory cytokines (IL-6, IL-8, IL-10, and IL-17) on wild-type colitis model

However, Paclik D et al. demonstrated that galectin-4 could induce T cell apoptosis by binding to the CD3 epitope at T cells surface on wild-type colitis model. Once binding to this epitope, galectin-4 promotes apoptosis of T cells in calpain-dependent manner and reduces the secretion of cytokines including IL-6, IL-8, IL-10, and IL-17, and then ameliorating the inflammation (Paclik et al., 2008a). Lately, another research found that the role of galectin-4 varied in different experimental colitis models (Mathieu et al., 2008). Based on the existing data, we can conclude that galectin-4 may exacerbate intestinal inflammation in TCR mutational colitis model, while ameliorate intestinal inflammation in wild-type colitis model (Fig. 3). Further studies are required to ascertain the universal role of galectin-4 in intestinal inflammation.

### Galectin-4 and cancer

Galectin-4 which has been detected in many cancers has association with the development and progression of pancreatic carcinoma, hepatocellular carcinoma, colorectal cancer (CRC), breast carcinoma, gastric cancer, and lung cancer (Rechreche et al., 1997; Hippo et al., 2001; Hayashi et al., 2013; Belo et al., 2013; Cai et al., 2014). However, it plays contradictory roles in different type of cancer cells. Furthermore, it has been detected in serum of some cancer patients (Kim et al., 2013; Cai et al., 2014; Barrow et al., 2011; Barrow et al., 2013). Up to now, although there is a number of published data regarding galectin-4 expression in cancer, the available information is remained limited. Among these cancers, only the role of galectin-4 in CRC development has been revealed explicitly.

#### The role of intracellular galectin-4 in cancer

In CRC, expression of galectin-4 was dramatically decreased compared to normal colon tissues and this condition promoted tumour progression and metastasis (Rechreche et al., 1997; Satelli et al., 2011; Kim et al., 2013). Lower expression of galectin-4 in CRC cells could induce increased cell proliferation, migration and motility. Galectin-4 was found to inhibit tumorigenesis of CRC cells through Wnt/β-catenin signaling pathway and IL-6/NF-κB/STAT3 signaling pathway (Satelli et al., 2011; Kim et al., 2013). In CRC cells, galectin-4 could cross-link Wnt signaling pathway proteins (APC, axin, and β-catenin), thereby stabilizing the destruction complex, and promoting degradation of β-catenin in cytoplasm (Satelli et al., 2011). Therefore, β-catenin could not enter into nucleus to activate the Wnt target genes, which results in the downregulation of cyclin D1, p21, and p15, and the inhibition of cell proliferation, migration, and motility. In addition, galectin-4 has been confirmed to downregulate IL-6, which in turn simultaneously inhibits the activation of nuclear factor-kappa B (NF-κB) and signal transducer and activator of transcription 3 (STAT3) in CRC (Kim et al., 2013; Lang et al., 2007). Upon suppressing of the IL-6/NF-κB/STAT3 signaling pathway, the level of vascular endothelial growth factor (VEGF), cyclooxygenase-2 (COX-2), and other genes involved in tumorigenesis would be downregulated, thereby inhibiting tumor progression.

A similar mechanism in pancreatic cancer was observed by Maftouh et al., 2014, where decreased levels of β-catenin were found in pancreatic cancer cells when in the presence of galectin-4. Their results coupled, therefore, the mechanism to a decreased Wnt signaling in the presence of galectin-4. Similarly, in hepatocellular cancer, the low level expression of galectin-4 contributes to increased metastasis and progression of the cancer (Cai et al., 2014). In addition, decreased expression of galectin-4 was observed in metastatic ileal carcinoids compared with primary carcinoid tumors in the ileum, indicating that galectin-4 may act as a tumor suppressor in ileal carcinoids (Rumilla et al., 2006). Conversely, in lung and gastric cancer, high level expression of galectin-4 was demonstrated to be an independent predictor for metastasis and correlated with poor clinical outcomes (Hayashi et al., 2013; Hippo et al., 2001). Taken together, galectin-4 acts as a tumor suppressor in CRC, pancreatic cancer, hepatocellular cancer, and ileal carcinoids, whereas galectin-4 functions as a tumor promoter in lung and gastric cancer.

#### The role of galectin-4 in serum of cancer patients in cancer

The free circulating level of galectin-4 in serum was significantly higher in patients with colon, hepatocellular, and breast cancer, in particular, those with metastasis (Barrow et al., 2011; Barrow et al., 2013; Kim et al., 2013; Cai et al., 2014). The expression level of galectin-4 was observed to be significantly increased up to 31-fold in the serum of colorectal cancer patients compared with healthy control group. Galectin-4 promotes cancer cell adhesion to vascular endothelial cells by interaction with the Thomsen-Friedenreich (TF) disaccharide on cancer-associated MUC1 (Barrow et al., 2011). As previously shown for galectin-3, this interaction causes MUC1 cell surface polarization, thus leading to exposure of underlying adhesion molecules that promote cancer-endothelium adhesion (Zhao et al., 2009), which indicates a metastasis-promoting effect. Furthermore, the interaction of galectin-4 with the vascular endothelium contributes to the increased circulating level of some cytokines and chemokines, including monocyte chemotatic protein 1 (MCP-1), granulocyte colony-stimulating factor (G-CSF) and IL-6 (Chen et al., 2014). Therefore, circulating level of galectin-4 might be a predictor for cancer patients, especially those with metastasis. However, no significant correlation between cancer stages and the level of circulating galectin-4 was observed in the serum of breast and colorectal cancer patients (Barrow et al., 2011).

## CONCLUSION REMARKS

Recent investigations of the molecular structure and physiological functions of galectin-4 have significantly increased our understanding of the potential roles of galectin-4 in some diseases, such as intestinal inflammation, tumors, etc. With further investigation of the effects and underlying mechanism, galectin-4 could be presented as a useful therapeutic target for killing bacteria, wound healing, and inhibiting tumorigenesis.

Many bacteria in human serum decorate their surfaces with diverse carbohydrate structures, and parts of these structures have similarities to human antigens, which leads to a difficult utilization of drugs to kill them directly. Galectin-4 may act as an innate defense lectin by recognizing the carbohydrates on the surface of *E.coli* O86 to kill the bacteria directly. In intestinal inflammation, galectin-4 has shown an inconsistent role in regulation of T cells, so the explicitly role should be further investigated in the future. Intracellularly, galectin-4 functions as a tumor suppressor, and its downregulation is an important event in the tumorigenesis of CRC, pancreatic cancer, and hepatocellular cancer, whereas it functions conversely in lung cancer and gastric cancer. Moreover, the expression of galectin-4 might be a biomarker in serum of colon and breast cancer patients. Although galectin-4 is not a universal and unambiguous marker in different types of cancers, it could be a helpful parameter in diagnosis of these tumors and clinical manifestations.
